# COVID-19 Vaccination Still Makes Sense: Insights on Pneumonia Risk and Hospitalization from a Large-Scale Study at an Academic Tertiary Center in Italy

**DOI:** 10.3390/microorganisms13081744

**Published:** 2025-07-25

**Authors:** Elena Azzolini, Brenda Lupo Pasinetti, Antonio Voza, Antonio Desai, Michele Bartoletti, Stefano Aliberti, Massimiliano Greco

**Affiliations:** 1 Department of Biomedical Sciences, Humanitas University, Via Rita Levi Montalcini 4, 20072 Milan, Italy; 2 IRCCS Humanitas Research Hospital, Via Manzoni 56, 20089 Milan, Italy

**Keywords:** COVID-19, vaccination, pneumonia, vaccine hesitancy, hospitalization

## Abstract

COVID-19 vaccines have revolutionized prevention and clinical management by reducing disease severity and mortality. However, their long-term impact on hospitalization is unclear. This retrospective study assessed whether vaccination status, timing, and number of vaccine doses influence the risk of hospitalization and COVID-19 pneumonia in a large cohort in Italy, several years after initial vaccine rollout. From 1 October 2023, to 2 February 2024, at Humanitas Research Hospital (Milan) and two affiliates, we recorded age, sex, comorbidities, vaccination status (number of doses and time since last dose), admission type (urgent vs. elective), and pneumonia diagnosis. Baseline health was quantified by the Charlson Comorbidity Index. Among 16,034 admissions (14,874 patients), vaccination data were available for 5743 cases: 40.8% were in the emergency setting and 59.2% were elective. Patients presented with pneumonia in 6.8% of cases. Laboratory results confirmed COVID-19 pneumonia occurred in 43.7% of pneumonia cases, with a 16.9% mortality. Patients with no vaccine dose had a higher proportion of COVID-19 pneumonia, while COVID-19 pneumonia rates were lower in individuals who had received more vaccine doses. There were no significant differences in COVID-19 pneumonia risk by timing of last vaccination. Moreover, hospitalized unvaccinated patients had overall more frequent emergency admissions (57.3%), while patients with three or more doses had about a ~40% emergency admission rate. COVID-19 positivity during hospitalization was highest in unvaccinated patients (90.7%) and declined with vaccination status. Vaccinated patients, especially those with multiple doses, had significantly lower COVID-19 pneumonia rates and emergency admissions. These findings suggest a possible protective effect of vaccination in modifying the clinical presentation and severity of illness among those who are hospitalized and support continued vaccination efforts for high-risk groups to reduce severe adverse outcomes.

## 1. Introduction

The diffusion of COVID-19 vaccines has significantly altered the approach to COVID-19 preventive management as well as its clinical course and management [1]. Although vaccines have shown substantial efficacy in decreasing serious illness and death in all age groups, their influence on specific outcomes such as pneumonia and hospitalization, especially in older populations, requires additional research [2,3,4].

Italy, similar to other countries, has implemented several types of COVID-19 vaccines, including mRNA-based vaccines and viral vector vaccines [2,3,5]. Some types of vaccine were subsequently withdrawn from the market, such as Vaxzevria from AstraZeneca, a viral vector vaccine, because of severe side effects, and in the last few years, mainly mRNA vaccines were employed in Italy [6]. While the mechanism of action of each vaccine type is distinct, and this can potentially lead to differences in immune response and protection against various manifestations of COVID-19 [7,8], a Cochrane review published in 2022 showed no difference between various types of vaccines and the development of side effects. Moreover, all the vaccines guaranteed a reduction in severe and critical disease [9].

Elderly patients represent a particularly vulnerable group, often with multiple comorbidities and age-related immune system decline. These factors may affect both the effectiveness of vaccination efficacy and susceptibility to pneumonia, whether caused by COVID-19 or other infections [10]. The relationship between vaccination status, vaccination type, and pneumonia risk in this population remains unclear.

Consequently, in elderly patients and in other high-risk populations, defining the best timing and number of vaccine doses is a critical factor. With the introduction of booster programs, it is essential to determine the optimal vaccination schedule to maintain protection, and continuous surveillance is necessary to monitor trends and adapt strategies to meet the evolving needs of the population based on their vaccination status [11].

We therefore conducted this study to evaluate whether the dose number and time of vaccination influence the risk of developing COVID-19 pneumonia and hospitalization in a large population of patients admitted to an academic tertiary center in Milan, Italy.

## 2. Materials and Methods

This retrospective observational study enrolled all consecutive patients admitted to Humanitas Research Hospital in Milan and two secondary hospitals from the same group in Italy, from 1 October 2023 to 2 February 2024.

All hospitalized patients with vaccination status were selected based on electronic health record data and anonymized before inclusion in the statistical analysis. This study received local IRB approval. We collected age, gender, comorbidities, vaccination status, number of doses, reason for admission, oxygen use, pneumonia data, clinical course, and outcomes.

COVID-19 positivity was defined according to the swab test results, using standardized terminology from laboratory and swab testing. Positive tests corresponded to standard RT-PCR detection of SARS-CoV-2 with high cycle threshold (Ct) values. Weakly positive tests included borderline RT-PCR positivity with low Ct values, and negative tests corresponded to undetectable viral RNA. In the study period, universal COVID-19 screening with swab testing was no longer routinely performed. Testing was reserved for high-risk patients and those presenting with respiratory symptoms, in accordance with regional health authority protocols.

This study followed the Strengthening the Reporting of Observational Studies in Epidemiology (STROBE) reporting guidelines.

### Statistical Analysis

Descriptive statistics were used to summarize demographic and clinical variables.

Categorical variables are reported as frequencies (percentages with 95% CIs) and continuous variables as means (with SDs) or medians (with interquartile ranges [IQRs] and 95% CIs) according to distribution. Groups were compared with Wilcoxon rank sum tests and with Pearson χ2 test (Fisher exact test where appropriate) for categorical variables.

The primary outcomes were the type of hospital admission (elective vs. emergency) and occurrence of pneumonia. COVID-19 infection status was categorized as positive, weakly positive, or negative based on swab test results. We used chi-square tests to assess associations between vaccination status and categorical outcomes (e.g., type of admission, COVID-19 test result, and pneumonia). Proportions and their 95% confidence intervals were estimated using binomial tests and visualized with bar plots and error bars.

Subgroup analyses were performed to explore pneumonia and COVID-19 pneumonia occurrence. Time since last vaccination was calculated and categorized in 180-day intervals, and its association with COVID-related pneumonia was evaluated using descriptive plots and chi-square tests. We analyzed data using R software, version 4.3 (R CoreTeam).

## 3. Results

From a total of 16,034 total hospital admissions out of 14,874 patients hospitalized over a period of 124 days from 1 October 2023 to 2 February 2024, we selected a total of 5743 patients presenting inclusion criteria.

A study flowchart is reported in Supplemental Material S1.

The 5743 patients with vaccination status data included 2348 (40.8%) emergency admissions and 3401 (59.2%) elective admissions. A total of 75 patients (1.3%) were not vaccinated, 70 (1.2%) had one dose of the vaccine, 520 (9.1%) had two doses, 3885 (67.7%) had three doses, 1026 (17.9%) had four doses, 155 (2.7%) had five doses, 16 (0.3%) had six doses, and 4 (0.06%) had more than six doses reported. Demographic characteristics and baseline data according to number of vaccine doses are reported in Table 1.

Unvaccinated patients had an overall higher proportion of emergency admissions, not limited to cases related to COVID-19 (57.3% [95% CI 45.5–68.5%]) compared with those who received one dose (44.3% [95% CI 35.6–56.6%]), two doses (37.9% [95% CI 33.7–42.2%]), or three doses (40.8% [95% CI 39.3–42.4%]). For patients who received more than three doses (up to seven or more), the proportion of emergency admissions remained relatively stable, at approximately 40%. (Table 2).

This is mirrored by the progressive increase in the proportion of overall elective hospital admissions according to vaccination status, rising from 42.7% (95% CI 31.5–54.6%) in unvaccinated patients, to 55.7% (95% CI 43.4–67.4%) in patients with one vaccination and peaking at about 60% with two or more vaccinations.

**Figure 1:**
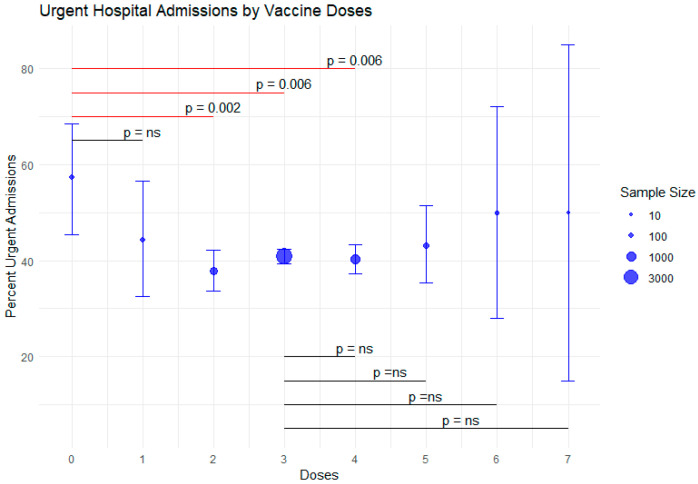
Overall emergency hospital admissions according to vaccination doses, with 95% CI. ns: Not significant.

In Figure 1, we plot the number of emergency hospital admissions by vaccination doses and compare different doses using a two-sample test for equality of proportions with continuity correction.

### COVID-19 Antigen Testing According to Vaccination Status

**Figure 2:**
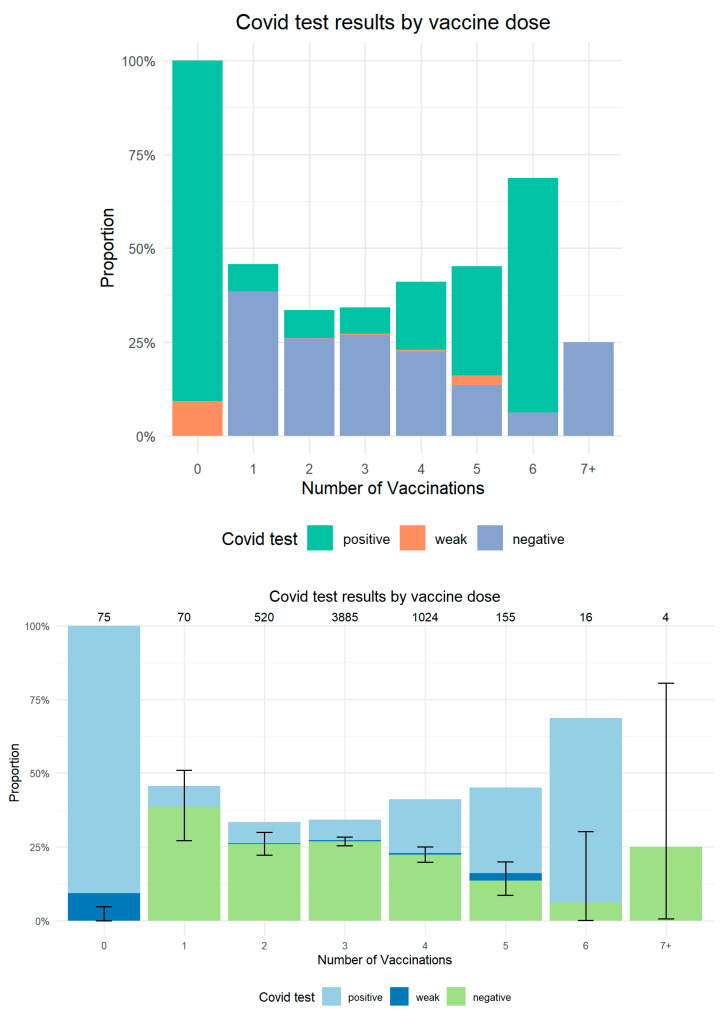
Positive COVID-19 nasopharyngeal swabs in hospitalized patients according to vaccination doses. Number of patients per group and 95% CI for negative swab results are reported.

A total of 2115 (36.9%) were hospitalized with a recorded COVID-19 swab test, 1537 (72.7%) were emergency admissions, and 578 (27.3%) were ordinary admissions. Of these patients, the large majority had a negative COVID-19 swab (68.9%), 29.5% had a positive COVID-19 swab, and 1.6% had a weak positive swab. Weakly positive swabs were clinically managed as positive patients and analyzed separately with positive tests because of the low occurrence. Figure 2 reports positive COVID-19 nasopharyngeal COVID-19 swabs according to vaccination status.

When stratifying by vaccination status, unvaccinated patients undergoing COVID-19 swab testing had a positive result in 90.7% and a weak positive result in 9.3%. There were no negative test results in unvaccinated patients. The percentage of positive tests dropped to 7.1% (95% CI 2.6–16.6%) in patients with one dose and 7.3% (95% CI 5.3–10.0%) in patients with two doses, progressing to 10.7% in patients with three or more doses. Among these, the proportion ranged from 6.9% (95% CI 6.2–7.8%) in patients with three doses and increased progressively to 62.5% (95% CI 35.9–83.7%) in patients with six doses (Figure 2). Unvaccinated patients had significantly more frequent positive COVID-19 tests than patients in all other categories (*p* < 0.0001).

There were 389 hospitalizations for COVID-19 and non-COVID-19 pneumonia (6.8% of 5749 admissions). In the whole population, COVID-19 pneumonia was diagnosed in 151 (38.8%) of pneumonia cases (2.6% of hospital admissions), leading to 26 deaths (16.9%). When considering COVID-19 pneumonia rates by vaccination status, unvaccinated hospitalized patients had COVID-19 pneumonia in 43.7% of cases vs. 6.9% of vaccinated patients with ≥3 doses. When assessing time since last vaccination and prevalence of COVID-19 pneumonia in this population, we did not find a statistically significant correlation (*p* = 0.45).

## 4. Discussion

We conducted a retrospective analysis of hospitalized patients according to their vaccination status for COVID-19 disease. We focused on a real-world population several years after the rollout of COVID-19 vaccines, allowing us to assess longer-term associations between vaccination status and hospital admissions. In general, we observed that unvaccinated individuals were more likely to require emergency hospital admission and had a significantly higher prevalence of positive or weakly positive COVID-19 test results compared with controls. Similarly, the proportion of hospitalized patients with COVID-19 pneumonia was significantly higher among unvaccinated individuals compared with those who had received vaccination.

As published in 2019 by the WHO, vaccination hesitancy is a serious problem all over the world; in particular, it is included among the ten threats to global health [12]. If this topic was relevant before the COVID-19 pandemic, it is now essential to face the problem to prevent or control the next pandemic.

During the different phases of the COVID-19 pandemic, there was a significant increase in vaccine hesitancy [13]. While the first dose of vaccine was generally considered as a necessary step to get over the most difficult period of the pandemic, skepticism grew around the effectiveness and safety of subsequent doses. Concerns about possible side effects led many individuals to decline additional vaccination [14].

The recommendation to re-vaccinate every 3–4 months, driven both by the rapid waning of neutralizing antibodies and by the continual emergence of immune-evasive variants (e.g., Omicron sub-lineages such as BA.2.86 and XBB), has added further concern. Unlike seasonal influenza—where a single annual shot is generally sufficient because antigenic drift is slower—SARS-CoV-2 currently demands a more frequent booster schedule, which many individuals perceive as burdensome and unnecessary.

Younger and more educated people showed greater trust in vaccines, especially for COVID-19, even if their confidence was reduced when they experienced side effects [15,16,17]. In addition, misinformation conveyed by the media, religion, and politics contributed to vaccination hesitancy among less educated people and those living in rural areas [18].

In our study, even if the percentage of unvaccinated patients is low, the difference in terms of hospitalization for COVID-19 pneumonia remains statistically significant. While it is difficult to assess how much COVID-19 vaccination can protect individuals from hospitalized patient data, the difference among the hospitalized population is evident. Moreover, some studies underlined the importance of COVID-19 vaccination to prevent and reduce long COVID and to protect organs, in particular heart, kidneys, and brain, from developing chronic diseases caused by COVID-19, or at least to reduce the risk and the impact of COVID-19 on organ function [19,20,21,22].

Vaccine hesitancy is also a key issue among health workers [23]. Several studies demonstrated that health care workers’ vaccination is helpful in preventing COVID-19 spread among patients, in particular in elderly and immunocompromised ones who are at particularly high risk [24,25,26,27,28,29]. Other studies underlined the importance of booster dose administration to maintain immune response [30,31,32]. In general, vaccine hesitancy can be a serious issue in future pandemics and could have a prominent impact on public health. The awareness of vaccine efficacy and safety among the general population should be developed and maintained to prevent a catastrophic impact on the public health system [13,33].

In this article, we have analyzed different aspects related to the impact of vaccination among hospitalized patients, focusing on elective vs. emergency admissions, risk of developing pneumonia, and vaccination timing. These are summarized in the following highlights and discussed below:

- Vaccination status is associated with the type of hospital admission required, with emergency hospital admission being more frequent in unvaccinated patients.
- Positive or weakly positive COVID-19 swabs were considerably more prevalent in unvaccinated patients requiring hospitalization.
- The proportion of COVID-19 pneumonia and overall pneumonia is higher in hospitalized patients with no COVID-19 vaccination compared with vaccinated individuals, with no influence of timing since last vaccination dose.

### 4.1. Vaccination Status and Hospital Admission Type

Our results show a significant association between vaccination status and the type of hospital admission. Unvaccinated patients had a higher frequency of emergency admissions compared with vaccinated individuals. This difference is likely not attributable solely to COVID-19 infection but may also reflect reduced access to—or lower utilization of—healthcare services among the unvaccinated population. This interpretation is further supported by the higher prevalence of hospitalizations for COVID-19-related pneumonia among unvaccinated patients, even when considering all causes of admission. This is consistent with the substantial body of literature that highlights the role of vaccination in reducing the severity of COVID-19 and consequently the need for emergency medical intervention [1,2,34,35,36].

Previous publications underlined the importance of booster dose administration to maintain immune response [29,30,31]. Booster vaccinations function to re-stimulate the immune system, elevating both antibody titers and memory B and T cell responses [37,38,39].

This is confirmed by our data, as shown in Supplementary Table S1, where prevalence of COVID-19 pneumonia in hospitalized patients reduces significantly after one dose, and this positive effect was maintained and even increased after the second and the booster dose of vaccine (considered as the third dose). While these findings on hospitalized patients cannot be translated to the whole population of vaccinated/unvaccinated individuals, they suggest that a booster dose may be useful to maintain long-term immunity, and support the notion that vaccination lowers the risk of severe disease, further highlighting its critical role in mitigating the public health impact [17,35].

### 4.2. Prevalence of Positive COVID-19 Testing

Our data also underscore the higher prevalence of positive or weakly positive COVID-19 test results in hospitalized patients with no previous vaccination compared with vaccinated patients. Previous studies show that patients who received at least two doses of vaccine had decreasing trends in COVID-19 case incidence [1].

While our data on hospitalized patients cannot directly confirm this, we demonstrated that when considering hospitalization, the unvaccinated patients exhibited a significantly higher proportion of positive test results, reinforcing the understanding that vaccination boosts immune protection, and this can have an effect on severe disease.

Recent studies underlined the positive impact of hybrid vaccination for both COVID-19 booster dose and influenza vaccine to prevent and reduce the risk of pneumonia and hospitalization for both the viruses [40] and underlined the efficacy of heterologous COVID-19 vaccination to prevent severe disease and to develop a stronger immunity against the virus ever more effectively than with the homologous vaccination [41].

For both the COVID-19 and influenza viruses, booster doses are mandatory to prevent infection and maintain immunity. Obviously, it is important to develop reformulated vaccines to target new subvariants to maintain strong protection [42,43]. Our findings, in conjunction with previous studies [9,44,45], emphasize the importance of vaccination as a strategy to control the spread of COVID-19, particularly among vulnerable populations such as the elderly and those with pre-existing health conditions.

### 4.3. Prevalence of COVID-19 and Non-COVID-19 Pneumonia

The proportion of hospitalization due to pneumonia, including COVID-19-related pneumonia, was notably higher among unvaccinated patients (Table 1), approximately six times higher than in vaccinated ones. Although the total number of pneumonia cases was relatively small, and the incidence of pneumonia in the overall population cannot be inferred from these data, our data confirm a trend towards higher hospitalization rates for pneumonia in unvaccinated individuals. This observation is consistent with the growing body of evidence that underscores the protective effects of COVID-19 vaccination in reducing the severity of infection and preventing complications such as pneumonia, which often lead to hospitalization [3,5].

Our data show a reduction in pneumonia in vaccinated people (even with only one dose). This is probably related to vaccines’ ability to enhance immune responses, thereby preventing viral replication and severe respiratory complications. An interesting point is the concept that hybrid immunity, derived from both vaccination and prior infection, may provide superior and longer-lasting protection compared with vaccine-induced immunity alone, as shown in the literature [46]. Nonetheless, the relatively small sample size of pneumonia cases in our cohort limits the ability to draw definitive conclusions, and further research with larger cohorts is necessary to better understand the relationship between vaccination status and pneumonia risk.

**Figure 3:**
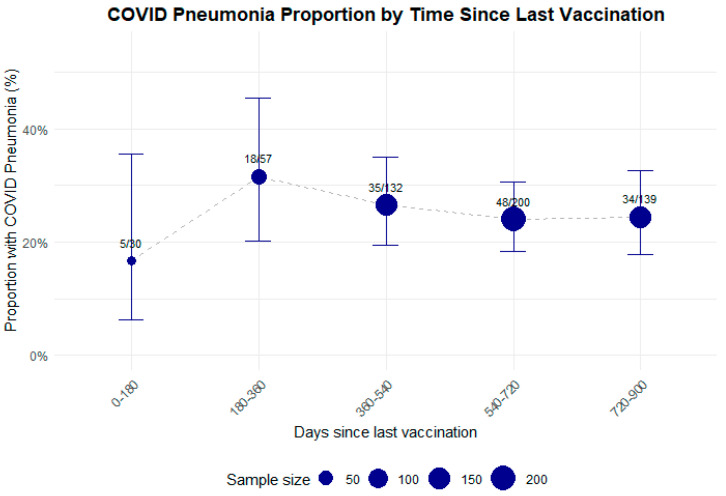
Proportion of patients with COVID-19 pneumonia according to vaccination timing (time since last vaccination).

The interval since the last vaccination was not associated with hospitalization for COVID-19 pneumonia in our population. This finding, as depicted in Figure 3, may seem in contrast with expectations that immunity might wane over time and that a longer interval since the last dose would lead to a higher risk of developing severe disease, including pneumonia. Nonetheless, a recent meta-analysis also suggested that the temporal decline in vaccine-induced antibodies after a full COVID-19 vaccination was moderate, maintaining protection against the development of COVID-19 pneumonia over time [47].

A possible explanation is that frail individuals—despite recent vaccination—may develop COVID-19 pneumonia because of a weaker immune response associated with age, comorbidities, or individual variability. As shown in other studies, vaccine effectiveness and immunogenicity tend to be lower and decline more rapidly in older adults [10,48,49,50,51]. Cancer, HIV, and comorbidities such as diabetes, hypertension, cardiovascular disease, and obesity also contribute to reduced vaccine efficacy [50,52,53]. Moreover, emerging variants of the virus or changes in viral characteristics over time may also contribute to the observed pneumonia outcomes, highlighting the dynamic nature of the pandemic and the challenges in maintaining vaccine efficacy against new strains.

In the Italian setting, people with ≥4 vaccine doses are not representative of the general ward population but of a policy-defined, exceptionally frail subgroup, as the Ministry of Health limits fourth and subsequent boosters to residents of long-term-care facilities, all adults ≥80 years, and 60–79-year-olds with major immunocompromised or other frailty conditions [54]. As a result, surveillance in November 2022 showed that—even with 65% second-booster coverage—people ≥ 80 years continued to post the highest national incidence of SARS-CoV-2 infection and hospitalization [55].

Our findings call for continued surveillance to monitor breakthrough infections and the evolving effectiveness of vaccines, especially in older adults and immunocompromised individuals.

### 4.4. Limitations

This study has several limitations. Despite the overall sample size being large, the distribution across categories was unbalanced, reflecting real-world prevalence. Consequently, patients with zero or more than four vaccine doses were underrepresented, which may have influenced statistical power for comparison involving these groups. We were also unable to perform multivariable regression analyses to adjust for potential confounders because of the highly unbalanced distribution of vaccination status and low event rates for certain outcomes. This limitation may affect the ability to isolate the independent effect of vaccination. Selective testing based on symptoms or risk profile may have introduced bias into the observed COVID-19 positivity rates across vaccination groups; nonetheless, this reflects the real-world scenario. Furthermore, unmeasured confounders such as frailty or prior infections may have influenced the results.

Due to the retrospective design of this study and the change in screening policy, detailed information about patients’ vaccination status was not fully available for all patients, reflecting real-world practice. In fact, of the 16,034 hospitalized patients in this study period analyzed, only 5743 had complete information about vaccination status and the number of doses. This can be related to different types of admissions. For instance, in the case of pneumonia or respiratory problems, it can be easier to find information about COVID-19 vaccinations. In other contexts, in which the cause of admission is different, such as neurologic diseases or trauma, these variables were more easily overlooked. Additionally, information on the type of vaccine administered was not available in our data, preventing vaccine-specific analysis.

## 5. Conclusions

In conclusion, our findings suggest that among hospitalized patients, COVID-19 vaccination is associated with a lower likelihood of emergency admission, confirmed COVID-19 diagnosis, and COVID-19 pneumonia. These results may reflect a protective role of vaccination in modifying the severity and clinical presentation of illness among those requiring hospitalization. While vaccination remains a cornerstone of public health strategies to mitigate the spread of COVID-19, the results also highlight the complexities of vaccine efficacy, particularly in high-risk populations and in the context of emerging variants.

Further research, particularly with larger sample sizes and longer follow-up periods, is needed to better understand these complex dynamics and optimize vaccination strategies to protect vulnerable populations.

## Figures and Tables

**Table 1:** Baseline characteristics according to number of vaccine doses.

	Overall	0	1–2	3	≥4	*P* for Difference
N *	5749	75	590	1458	623	
Age (mean (SD))	66.52 (15.75)	70.32 (14.07)	58.83 (17.39)	69.17 (15.55)	73.70 (14.38)	<0.001
Sex = Male (*n*, %)	3289 (57.2)	33 (44.0)	319 (54.1)	863 (59.2)	358 (57.5)	0.764
CCI * (mean (SD))	1.84 (2.27)	2.75 (3.02)	1.62 (2.23)	2.46 (2.57)	2.40 (2.31)	0.881
Hypertension (*n*, %)	3007 (52.3)	45 (60.0)	213 (36.1)	836 (57.3)	408 (65.5)	<0.001
COVID-19 (*n*, %)						<0.001
Negative	1458 (68.9)	0 (0.0)	162 (78.6)	1458 (100.0)	0 (0.0)	
Positive	623 (29.5)	68 (90.7)	43 (20.9)	0 (0.0)	623 (100.0)	
Weak	34 (1.6)	7 (9.3)	1 (0.5)	0 (0.0)	0 (0.0)	
Pneumonia (*n*,%)	389 (6.8)	17 (22.7)	35 (5.9)	202 (13.9)	151 (24.2)	<0.001
COVID-19 Pneumonia (*n*, %)	157 (43.7)	17 (100.0)	10 (33.3)			

* N: total patients; CCI: Charlson Comorbidity Index.

**Table 2:** Type of hospitalization according to doses.

Vaccine Doses	Frequency	Emergency Admission (%)	95% CI Emergency Admission	Standard Admission (%)	95% CI Standard Admission
0	75	57.3	[45.4, 68.5]	42.7	[31.5, 54.6]
1	70	44.3	[32.6, 56.6]	55.7	[43.4, 67.4]
2	520	37.9	[33.7, 42.2]	62.1	[57.8, 66.3]
3	3885	40.8	[39.3, 42.4]	59.2	[57.6, 60.7]
4	1024	40.3	[37.3, 43.4]	59.7	[56.6, 62.7]
5	155	43.2	[35.4, 51.4]	56.8	[48.6, 64.6]
6	16	50	[28.0, 72.0]	50	[28.0, 72.0]
7+	4	50	[15.0, 85.0]	50	[15.0, 85.0]

## Data Availability

The raw data supporting the conclusions of this article will be made available by the authors on request.

## Supplementary Materials

The following supporting information can be downloaded at: https://www.mdpi.com/article/10.3390/microorganisms13081744/s1, Supplemental Material S1: Study flow chart; Supplementary Table S1: Percentage of positive COVID-19 swabs among vaccinated and unvaccinated patients.

## Abbreviations

The following abbreviations are used in this manuscript:

CCI	Charlson Comorbidity Index
CI	Confidence interval
IQR	Interquartile ranges
SD	Standard deviation
