# Supplementary material for: COVID-19 Vaccination Still Makes Sense: Insights on Pneumonia Risk and Hospitalization from a Large-Scale Study at an Academic Tertiary Center in Italy
Source: Microorganisms. 2025 Jul 25;13(8):1744. doi: 10.3390/microorganisms13081744 (PMC12388399; doi:10.3390/microorganisms13081744)
Supplement: Supplementary file 1 [file microorganisms-13-01744-s001.zip › microorganisms-3727294-supplementary.pdf]

Supplemental material S1

Study flow chart

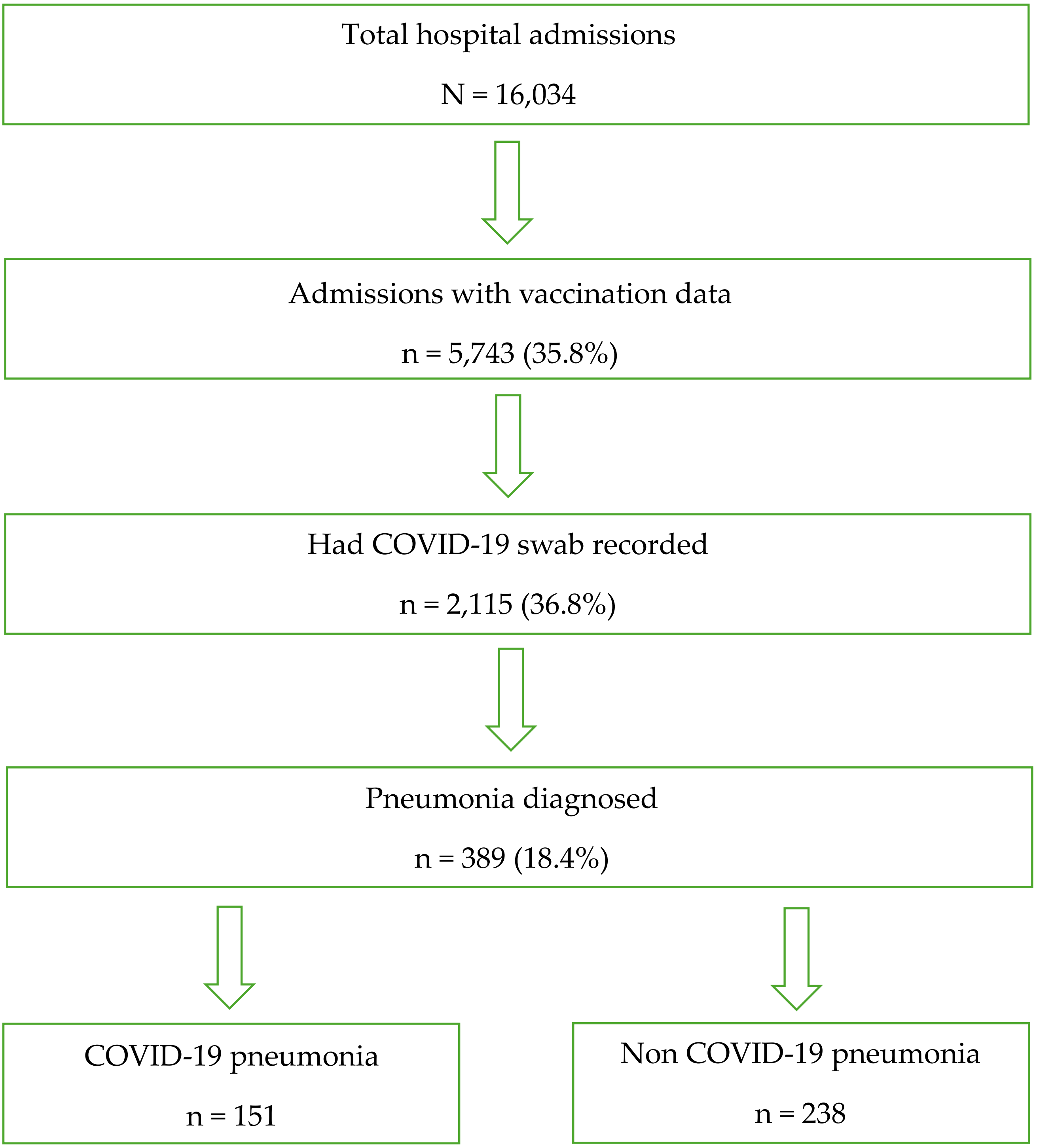

Supplemental material S2

Table S1. Percentage of positive COVID-19 swabs among vaccinated and unvaccinated patients.

Doses	Total Admission	COVID Positive	Positivity rate (%)
**0**	75	**68**	90.7
**1**	70	**5**	7.1
**2**	520	**38**	7.3
**3**	3885	**271**	7.0
**4**	1024	**186**	18.2
**5**	155	**45**	29.0
**6**	16	**10**	62.5
**7**	4	**0**	0.0
